# Construction of a network describing asparagine metabolism in plants and its application to the identification of genes affecting asparagine metabolism in wheat under drought and nutritional stress

**DOI:** 10.1002/fes3.126

**Published:** 2018-02-25

**Authors:** Tanya Y. Curtis, Valeria Bo, Allan Tucker, Nigel G. Halford

**Affiliations:** ^1^ Plant Sciences Department Rothamsted Research Harpenden Hertfordshire UK; ^2^ College of Engineering, Design and Physical Sciences Brunel University London Uxbridge Middlesex UK; ^3^ Present address: Cancer Research UK Cambridge Institute University of Cambridge Li Ka Shing Centre Robinson Way Cambridge UK

**Keywords:** asparagine metabolism, asparagine synthetase, glutamine synthetase, stress responses, systems approaches

## Abstract

A detailed network describing asparagine metabolism in plants was constructed using published data from Arabidopsis (*Arabidopsis thaliana*) maize (*Zea mays*), wheat (*Triticum aestivum*), pea (*Pisum sativum*), soybean (*Glycine max*), lupin (*Lupus albus*), and other species, including animals. Asparagine synthesis and degradation is a major part of amino acid and nitrogen metabolism in plants. The complexity of its metabolism, including limiting and regulatory factors, was represented in a logical sequence in a pathway diagram built using yED graph editor software. The network was used with a Unique Network Identification Pipeline in the analysis of data from 18 publicly available transcriptomic data studies. This identified links between genes involved in asparagine metabolism in wheat roots under drought stress, wheat leaves under drought stress, and wheat leaves under conditions of sulfur and nitrogen deficiency. The network represents a powerful aid for interpreting the interactions not only between the genes in the pathway but also among enzymes, metabolites and smaller molecules. It provides a concise, clear understanding of the complexity of asparagine metabolism that could aid the interpretation of data relating to wider amino acid metabolism and other metabolic processes.

## 1 INTRODUCTION

Free asparagine plays a central role in nitrogen storage and transport in many plant species due to its relatively high ratio of nitrogen to carbon and its unreactive nature (Lea, Sodek, Parry, Shewry, & Halford, 2007). It accumulates to high concentrations during processes such as seed germination and in response to a range of abiotic and biotic stresses (Forde & Lea, 2007; Halford, Curtis, Chen, & Huang, 2015; Lea & Azevedo, 2007; Lea et al., 2007). For example, free asparagine, together with proline and glycine betaine (an N‐trimethylated amino acid), accumulates in *Hordeum* species in response to salt stress (Garthwaite, von Bothmer, & Colmer, 2005), while there is a 15‐ and 28‐fold rise in the concentration of free asparagine and proline, respectively, in drought‐stressed pearl millet (*Pennisetum glaucum*) (Kusaka, Ohta, & Fujimura, 2005). Asparagine is the predominant free amino acid in potato tubers (Halford et al., 2012) and its concentration increases further in some varieties in response to severe drought stress (Muttucumaru, Powers, Elmore, Mottram, & Halford, 2015). It can also become the predominant free amino acid in cereal grains under some stress conditions. Furthermore, there is evidence from several studies that free asparagine concentration varies considerably in the grain of both wheat (*Triticum aestivum*) and rye (*Secale cereale*) sourced from different locations or grown in different years or under different crop management regimes, showing that asparagine metabolism is responsive to multiple environmental and crop management factors (Baker et al., 2006; Claus et al., 2006; Curtis et al., 2009, 2010; Curtis et al., 2016; Postles, Powers, Elmore, Mottram, & Halford, 2013; Postles et al., 2016; Martinek et al., 2009; Taeymans et al., 2004). The fact that free asparagine and other free amino acids accumulate to high concentrations in plant tissues in response to stress is an example of how stress can have profound effects on crop composition (Halford et al., 2015).

Free asparagine concentration also responds to nutrient availability: For example, it has been shown to correlate positively with nitrogen availability in the grain of barley (*Hordeum vulgare*) (Winkler & Schön, 1980), wheat (Martinek et al., 2009), and rye (Postles et al., 2013, 2016), while deficiencies in other minerals become important when there is a plentiful supply of nitrogen (reviewed by Lea et al., 2007). Sulfur deficiency in particular can cause a massive (up to 30‐fold) increase in the accumulation of free asparagine in wheat, barley, and maize (*Zea mays*) (Baudet et al., 1986; Curtis et al., 2009; Granvogl, Wieser, Koehler, von Tucher, & Schieberle, 2007; Muttucumaru et al., 2006; Shewry, Franklin, Parmar, Smith, & Miflin, 1983). Rye responds in similar fashion in response to severe sulfur deficiency in pot experiments (Postles et al., 2016) but is less responsive under field conditions (Postles et al., 2013). Consistent with this, asparagine synthetase gene expression in wheat and rye has been shown to increase under sulfur‐limited growth conditions (Byrne et al., 2012; Gao et al., 2016; Postles et al., 2016), a response that appears to involve the protein kinase, TaGCN2 (Byrne et al., 2012).

The changes in free asparagine concentration in grains and tubers in response to stress and nutrition suggest that the regulation of asparagine metabolism has implications for crop yield and stress resistance. However, the issue that has stimulated interest in asparagine metabolism and accumulation more than any other is the role of free asparagine in the formation of acrylamide, a Group 2A carcinogen, during high‐temperature cooking and processing. Acrylamide formation affects fried and roasted potato products, bread and crisp‐bread, biscuits, breakfast cereals, coffee, chocolate, and other popular foods (EFSA Panel on Contaminants in the Food Chain (CONTAM), 2015).

If crop and agronomic approaches are to contribute to addressing this problem, ways of reducing the accumulation of free asparagine in grains, beans, and tubers, and of making it less sensitive to environmental factors, will have to be developed. This will require a comprehensive understanding of the factors that control asparagine metabolism and how free asparagine accumulation is affected by other areas of plant metabolism and the environment. Systems biology and mathematical modeling have been used in a variety of applications to elucidate and explain the mechanisms of complex metabolic and signaling networks (Breitling, Donaldson, Gilbert, & Heiner, 2010), and this study applied this approach to describe asparagine metabolism in plants. A network was constructed using information available in the literature and publicly available databases, comprising genes, enzymes, transcription factors, and regulatory proteins, as well as small molecules such as asparagine itself, other free amino acids, and energy molecules. Most of the information was derived from studies on Arabidopsis (*Arabidopsis thaliana*), with additional information from a variety of species, but the applicability of the network to a major crop species, wheat (*Triticum aestivum*), was demonstrated through the analysis of multiple wheat microarray studies using a Unique Network Identification Pipeline (UNIP) developed previously (Bo et al., 2014). This analysis identified subnetworks of genes and was extended to detect the most predictive genes for unstressed, drought‐stressed, and sulfur and nitrogen deficiency conditions; in other words, those genes that were most closely associated with each stress.

## 2 MATERIALS AND METHODS

A network of asparagine metabolism was constructed based on articles from the literature (Baena‐González, Rolland, Thevelein, & Sheen, 2007; Curien et al., 2009; Gaufichon, Reisdorf‐Cren, Rothstein, Chardon, & Suzuki, 2010; Hey, Mayerhofer, Halford, & Dickinson, 2007; Hsieh, Lam, & Coruzzi, 1996; Hummel, Rahmani, Smeekens, & Hanson, 2009; Lam et al., 1995; Lima & Sodek, 2003; Nikiforova et al., 2006; Piotrowski & Volmer, 2006; Romagni & Dayan, 2000; Sato, Arita, Soga, Nishioka, & Tomita, 2008; Todd et al., 2008; Wan, Shao, Shan, Zeng, & Lam, 2006; Weltmeier et al., 2009) and reviews of publicly available databases, including: http://www.arabidopsisreactome.org/; http://www.ebi.ac.uk/biomodels-main/; http://string-db.org/; http://www.arabidopsis.org/; and http://www.brenda-enzymes.org/. The network was constructed using yED Graph Editor Version 3.2.0.1 (yWorks, Tübingen Germany). This program, which is available free from https://www.yworks.com/downloads#yEd, provided enough freedom to construct the network with genes, enzymes, and small molecules. Most of the information that was used related to work done with Arabidopsis (*Arabidopsis thaliana*); however, data from other plant species, including maize (*Zea mays*), wheat (*Triticum aestivum*), pea (*Pisum sativum*), soybean (*Glycine max*), and lupine (*Lupus albus)* and others, as well as some animal and human data were also used.

To test the genes identified in the asparagine metabolism network under different stress conditions, and to explore the applicability of the network to wheat, the UNIP pipeline (Bo et al., 2014) was applied to a set of publicly available wheat microarray transcriptomic data (Table 1) in two different scenarios: Case 1, to explore the underlying mechanisms operating in all stress conditions but not in nonstress conditions; Case 2, to identify the unique mechanism underlying each type of stress but not operating under nonstress conditions. The raw data were preprocessed using the Robust Multi‐Array Average (RMA) (Hell & Bergmann, 1990) and the subset of genes identified in the asparagine metabolism network were selected to proceed. Independently of the case scenario, glasso (Friedman, Hastie, & Tibshirani, 2008) was also applied to construct each gene regulatory network and select the unique connections (edges that appear only in the network under consideration but not in the others). The bnlearn package was used in R (Scutari, 2010) to build Bayesian networks using the hill‐climbing technique, taking advantage of the inference feature to calculate the prediction accuracy for each gene.

**Table 1:** ID, number of samples, and descriptions of wheat datasets used in this study. Datasets 1 to 12 are stress enriched while the remaining are nonstress

No	Study ID	Samples	Description
1	E‐GEOD‐42214	12	Wheat drought responses
2	E‐MTAB‐903	30	Transcription profiling by array of winter wheat grown using different agricultural practices
3	E‐MTAB‐963	36	Transcription profiling by array of wheat leaves in response to the fungal toxin ToxB from *Pyrenophora tritici‐repentis*
4	E‐GEOD‐30436	24	Transcriptome profiling of reproductive stage flag leaves of wheat from drought susceptible parent WL711, drought‐tolerant parent C306, and drought‐susceptible and drought‐tolerant RIL bulks in irrigated and drought condition
5	E‐GEOD‐31759	27	Drought stress in wheat at grain filling stage
6	E‐MEXP‐971	60	Transcription profiling of two highly salt‐tolerant wheat lines, their parental lines, and a salt‐sensitive line in salt stress and control growth conditions
7	E‐MEXP‐1415	36	Transcription profiling time series of leaves from winter wheat grown under S‐ and N‐deficient conditions
8	E‐MEXP‐1193	32	Transcription profiling time series of wheat cv. Hereward grown under control, hot, dry, and hot and dry conditions to illustrate the importance of developmental context in interpretation
9	E‐MEXP‐1523	30	Transcription profiling of heat‐tolerant and susceptible strains of wheat after exposure to heat stress
10	E‐MEXP‐1669	72	Profiling of six winter wheat varieties grown under different nitrogen fertilizer levels
11	E‐GEOD‐12936	12	Transcription profiling of the effect of silicon on wheat plants infected or uninfected with powdery mildew
12	E‐GEOD‐11774	42	Transcription profiling of wheat cultivars after cold treatment
13	E‐GEOD‐4935	78	Transcription profiling of wheat—expression level polymorphism study: 39 genotypes and two biological replicates
14	E‐GEOD‐6027	21	Transcription profiling of wheat meiosis and microsporogenesis in hexaploid bread wheat
15	E‐GEOD‐9767	16	Transcription profiling of wheat to identify genotypic differences in water soluble carbohydrate metabolism in stem
16	E‐GEOD‐12508	39	Transcription profiling of wheat development
17	E‐GEOD‐5939	72	Transcription profiling of wheat—expression level polymorphism study: 36 genotypes and two biological replicates from SB location
18	E‐GEOD‐5942	76	Transcription profiling of wheat expression level polymorphism study parentals and progenies from SB location

## 3 RESULTS

A network describing asparagine metabolism was constructed initially on the basis of original studies in Arabidopsis under different physiological conditions conducted by Lam et al. (1995). More details were added from a wider literature search and database review, including genes and enzymes from other plant species. The final network consisted of 212 nodes (genes, enzymes, or molecules; Table S1) and 246 edges (reactions between nodes). It is provided as a Figure S1 because it is too large to include as a standard figure.

The main enzymes identified as being involved in asparagine metabolism were as follows: asparagine synthetase (ASN), glutamine synthetase (GS), glutamate dehydrogenase (GDH), ferredoxin‐dependent glutamate synthase (Fd‐GOGAT: GLU1 and GLU2), NADH‐dependent glutamate synthase (NADH‐GOGAT), aspartate amino transferase (AspAT), and glutamate decarboxylase (GAD) (Figure S1).

### 3.1 Regulation of asparagine synthetase gene expression

Asparagine is synthesized from glutamine and aspartate by glutamine‐dependent asparagine synthetase. The asparagine synthetase enzyme also needs adenosine triphosphate (ATP) and Mg^2+^ for the transfer of an amino group from glutamine to aspartate and the release of asparagine. Asparagine synthetase cDNAs were first isolated by Tsai and Coruzzi (1990) from pea (*Pisum sativum*), and were shown to encode two enzymes, AS1 and AS2. A distinct asparagine synthetase gene, *AS*, was subsequently shown to be induced in harvested asparagus spears in response to carbohydrate stress (Davies & King, 1993). Arabidopsis is now known to contain three asparagine synthetase genes, *AtASN1, AtASN2,* and *AtASN3*, whereas potato has two: *StASN1*, which is expressed at high levels in the tuber, and *StASN2*, which is expressed throughout the plant (Chawla, Shakya, & Rommens, 2012). Maize, wheat, and barley, on the other hand, all have four asparagine synthetase genes that are active in different parts of the plant (Avila‐Ospina, Marmagne, Talbotec, & Krupinska, 2015; Duff et al., 2011; Gao et al., 2016; Todd et al., 2008), and this may be the pattern for all cereal species. Of the four genes in wheat, Gao et al. (2016) showed *TaASN2* to be the most highly expressed in the grain. Indeed, expression of *TaASN2* in the grain (both embryo and endosperm) dwarfed expression of any of the four genes in any other tissue. However, *TaASN1* was the most responsive to nitrogen availability and sulfur deficiency, and had previously been shown to respond to salt stress, osmotic stress, and ABA (Wang, Liu, Sun, & Zhang, 2005).

In Arabidopsis, *AtASN1*,* AtASN2,* and *AtASN3* are expressed in different tissues and differentially regulated by stress stimuli, light, and sucrose (Lam, Hsieh, & Coruzzi, 1998). Light, for example, represses expression of *ASN1* in a phytochrome‐dependent manner, whereas expression of *ASN2* is extremely low in the dark but rapidly induced by light treatment. Overall, asparagine accumulates in the tissues of dark‐adapted Arabidopsis plants (Lam, Peng, & Coruzzi, 1994). The expression of both *AtASN1* and *AtASN2* is also affected by the supply of organic nitrogen in the form of glutamate, glutamine, or asparagine (Lam et al., 1998). However, *AtASN1* expression in tissue culture is repressed by sucrose feeding, whereas *AtASN2* expression is not. The signaling pathway through which sucrose affects *AtASN1* in Arabidopsis is shown in the top right hand section of Figure S1. Note that when sucrose is supplied to plants in culture, it may be cleaved by invertases to glucose and fructose or by sucrose synthase to UDP‐glucose and fructose. It is often unclear which of these molecules is initiating a response. However, it is now established that the sugar‐sensing signaling pathway in plants involves a protein kinase, sucrose nonfermenting‐1 (SNF1)‐related protein kinase‐1 (SnRK1) (reviewed by Hey, Byrne, & Halford, 2010), and reporter gene expression driven by the Arabidopsis *AtASN1* promoter (also referred to as the dark‐inducible‐6 (*DIN6*) promoter) has been shown to be greatly increased by overexpression of SnRK1 (Baena‐González & Sheen, 2008; Baena‐González et al., 2007; Confraria, Martinho, Elias, Rubio‐Somoza, & Baena‐González, 2013). There are two SnRK1s in Arabidopsis; these are shown as SnRK1.1 and SnRK1.2 in Figure S1, but they are also known as AKIN10 and AKIN11. The signaling pathway also involves the S1 class of bZIP transcription factors: low sucrose induces asparagine synthetase gene expression *via* AtbZIP11, while high levels of sucrose induce expression of genes encoding AtbZIP9, AtbZIP10, AtbZIP25, and AtbZIP63, all of which inhibit asparagine synthetase gene expression (Hummel et al., 2009).

Activity of AtSnRK1 and hence potentially its induction of *AtASN1* gene expression is also regulated posttranslationally by phosphorylation by its upstream kinase, SnRK1‐activating kinase (SnAK), which is encoded by two genes, *AtSnAK1* and *AtSnAK2* (Hey et al., 2007). The relationship between AtSnRK1 and AtSnAK1/2 is complex, involving autophosphorylation of AtSnAK1/2 and cross‐phosphorylation between the two protein kinases (Crozet et al., 2010).

Another protein kinase, general control nonderepressible‐2 (GCN2), has been shown to affect *TaASN1* gene expression in wheat, and was therefore included in the network. GCN2 phosphorylates the α subunit of translation initiation factor eIF2 (eIF2α). In fungi, it is activated in response to a reduction in free amino acid concentrations, and maintains the balance between free amino acids and proteins (reviewed by Hinnebusch, 1992; Halford, 2006). An Arabidopsis homologue has been shown to be activated in response to herbicides that inhibit amino acid biosynthesis (Zhang, Dickinson, Paul, & Halford, 2003; Zhang et al., 2008), as well as multiple stress stimuli, including purine deprivation, UV light, cold shock, wounding, pathogen infection, methyl jasmonate, salicylic acid, and cadmium exposure (Lageix et al., 2008). Overexpression of the wheat homologue, *TaGCN2*, in transgenic wheat resulted in significant decreases in total free amino acid concentration in the grain, with free asparagine concentration in particular being much lower than in controls (Byrne et al., 2012). Expression of *TaASN1* and genes encoding cystathionine γ‐synthase and sulfur‐deficiency‐induced‐1 (*SDI1*) all decreased significantly, while that of a nitrate reductase gene increased. Sulfur deficiency‐induced activation of *TaASN1* and *SDI1* occurred in wild‐type plants but not in *TaGCN2* overexpressing lines (Byrne et al., 2012). GCN2 activity has also been linked with asparagine synthetase gene expression and sulfur metabolism in mammalian systems: both phosphorylation of eIF2α and expression of asparagine synthetase have been shown to be higher in liver cells of rats fed a diet deficient in sulfur‐containing amino acids than of well‐nourished rats (Sikalidis & Stipanuk, 2010).

An additional regulatory mechanism of *AtASN1* gene expression that was included in the network involves the homeobox‐leucine zipper protein 22 (HAT22) (Thum et al., 2008). Genes encoding two other important metabolic enzymes, pyruvate phosphate dikinase (PPDK) and alanine‐glyoxylate aminotransferase (AGT), are also connected to *HAT22*. This suggests that *HAT22* may be involved in coordinating the regulation of genes in three metabolic processes: amino acid metabolism, carbohydrate metabolism, and glycolysis. *HAT22* shows additional regulatory edge connections to four other genes (Figure S1), *WRKY23*,* SINA,* a light regulated but otherwise uncharacterized gene protein (accession number At3g26740), and a gene annotated only as an expressed protein (accession number At3g20340). The mechanism of regulation of these genes by HAT22 is not described in detail in the literature.

### 3.2 Role of the glutamine loop in asparagine metabolism

As asparagine is synthesized by transfer of an amino group from glutamine to aspartate, the synthesis of asparagine is likely to be dependent on the availability of glutamine and aspartate and this has been demonstrated experimentally for the human enzyme (Van Heeke & Schuster, 1989). In plants, glutamine and aspartate are derived from nitrogen assimilation, and this is represented in the top left corner of Figure S1.

Nitrogen is taken up by plants in the form of inorganic nitrate, which is reduced to nitrite by nitrate reductase (NR). Nitrite is then further reduced to ammonium by nitrite reductase (NiR), and the ammonium is assimilated into amino acids in a process that is catalyzed initially by glutamine synthetase (GS) and glutamate synthase (also known as glutamine oxoglutarate aminotransferase, or GOGAT). Glutamine synthetase catalyzes the ATP‐dependent condensation of glutamate and ammonia to form glutamine (Bernard & Habash, 2009), while GOGAT isoenzymes, NADH‐GOGAT and Fd‐GOGAT, catalyze the transfer of the amido nitrogen of glutamine to 2‐oxoglutarate to make glutamate, using NADH/NADPH or ferredoxin as reductants.

There are two subspecies of GS with different cellular localization: GS1 in the cytosol and GS2 in plastids. The enzyme is represented in Figure S1 as being encoded by genes *GSe2*,* GSe1*,* GS1a*,* GS1b*, and *GS1c*, all of which are expressed in the leaves and upper parts of plants, as well as *GSr1* and *GSr2*, which are expressed in the roots. The two‐step reaction has been described by Unno et al. (2006), but a simplified version is included in Figure S1.

Glutamate synthase (Fd‐GOGAT) activity is affected by light and sucrose supply in leaves (Coschigano, Melo‐Oliveira, Lim, & Coruzzi, 1998; Singh, 1999), and these are shown as major regulatory factors in the pink oval above the Fd‐GOGAT gene *GLU1* in Figure S1. Arabidopsis contains a second gene, *GLU2*, encoding Fd‐GOGAT, and while *GLU1* is expressed at highest levels in the leaves and is significantly induced by light or sucrose, *GLU2* is expressed predominantly in the roots (Coschigano et al., 1998). Salt stress has been shown to affect Fd‐GOGAT activity and protein level in both tomato (Berteli et al., 1995) and potato (Teixeira & Fidalgo, 2009).

The GS‐GOGAT pathway is the primary route for ammonia assimilation in plants (reviewed by Lea & Azevedo, 2007) but there is another route *via* glutamate dehydrogenase (GDH) (Figure S1). This enzyme catalyzes the NADPH‐dependent conversion of ammonia and 2‐oxoglutarate to glutamate. It may function in the direction of glutamate catabolism in dark‐treated or sugar‐starved plants, and expression of the *GDH1* gene increases in Arabidopsis under those conditions (Melo‐Oliveira, Oliveira, & Coruzzi, 1996). However, *GDH1* expression is also induced in the light by supplying ammonia, and under these conditions (plentiful carbon from photosynthesis as well as nitrogen) the enzyme may function in the direction of glutamate biosynthesis (Melo‐Oliveira et al., 1996).

### 3.3 Aspartate kinase and asparaginase

The enzyme asparaginase appears in the network for the obvious reason that it catalyzes the hydrolysis of the amide group of asparagine to release aspartate and ammonia, the latter being reincorporated into amino acid metabolism by glutamine synthetase. It may therefore play an important role in the supply of nitrogen to sink tissues through the processing of incoming, transported asparagine, and in the remobilization of free asparagine that has accumulated in response to nutrient deficiency or stress. Two types of asparaginase enzyme have been described in plants, differentiated according to whether or not their catalytic activity is potassium‐dependent. In Arabidopsis, potassium‐independent asparaginase is encoded by gene At5g08100 and potassium‐dependent asparaginase by At3g16150 (Bruneau, Chapman, & Marsolais, 2006). The relatively high catalytic efficiency of the potassium‐dependent enzyme suggests that it may metabolize asparagine more effectively (Bruneau et al., 2006). A third gene, At4g0050590, has been annotated as an asparaginase gene but has not been characterized in detail. There is evidence from soybean that asparaginase may be induced by low‐temperature stress (Cho et al., 2007).

Another enzyme, aspartate amino transferase (AspAT), also catalyzes a reaction that produces aspartate, thereby potentially making aspartate available for asparagine synthesis, but in this case it is a reversible reaction between oxaloacetate and glutamate, and it produces α‐ketoglutarate in addition to aspartate. Arabidopsis contains multiple isoenzymes of AspAT, localized in the cytosol, chloroplast, mitochondria, and peroxisomes (Schultz, Hsu, Miesak, & Coruzzi, 1998). They are encoded by genes *ASP1* (Heazlewood et al., 2004; Millar, Sweetlove, Giege, & Leaver, 2001; Wilkie & Warren, 1998), *ASP2* (Brauc, De Vooght, Claeys, Hofte, & Angenon, 2011), *ASP3* (Funakoshi et al., 2008), and *ASP4* (Theologis et al., 2000). Of these, the most important for nitrogen transport appears to be *ASP2*, which encodes a cytosolic enzyme because mutant plants with a defective *ASP2* gene show an 80% reduction in the levels of aspartate being transported in the phloem in the light and a 50% reduction in the dark (Schultz et al., 1998). Thus, cytosolic AspAT may control the synthesis of aspartate for nitrogen transport in the light, with the aspartate pool that it provides being available for conversion into asparagine in the dark (Schultz et al., 1998).

Another enzyme that affects aspartate availability is aspartate kinase (also known as aspartokinase), but in this case it competes with asparagine synthetase for aspartate, thereby potentially reducing the amount of aspartate available for asparagine synthesis. Aspartate kinase is encoded by genes *AK‐HSDH II*,* AK1*,* AK3*,* AK2iso,* and *AK‐HSDH I*, which feature in Figure S1 in the lower middle section. The enzyme exists as a monofunctional aspartate kinase (two isoforms: AK1 and AK2), and bifunctional aspartate kinase‐homoserine dehydrogenase (two isoforms: AK‐HSDH I and AK‐HSDH II) (Curien et al., 2009). It catalyzes the phosphorylation of aspartate, which is the first step in the biosynthesis of the other ‘aspartate family’ amino acids: methionine, lysine, and threonine.

### 3.4 Applicability of the network to wheat

In order to strengthen the findings displayed in the network shown in Figure S1, and test its applicability to a major crop, the responses of key genes in the network to different stresses were investigated using existing, publicly available, wheat transcriptomic data. Given a set of data from studies of wheat under different conditions, the Unique Network Identification Pipeline (UNIP) described in detail by Bo et al. (2014) identifies subnetworks that uniquely appear in the condition under consideration. UNIP was applied to further explore and strengthen the network in Figure S1 from a computational point of view. Eighteen independent wheat datasets (Table 1) were downloaded from the ArrayExpress database (Parkinson et al., 2009) in order to do this. Note that UNIP does not identify all links involved in an underlying biological mechanism; rather only those that uniquely exist in the treatment/condition of interest. A link‐by‐link comparison of the literature‐derived network and the UNIP‐derived networks is therefore not appropriate and was not applied in this study.

Given the raw structure of the data, the Robust Multi‐Array Average (rma) expression measure (Gautier, Cope, Bolstad, & Irizarry, 2004) was applied as a preprocessing step. In each dataset, the 121 Affymetrix IDs of the genes that had been identified as being involved in asparagine metabolism (Table S1) were selected. Once these reduced datasets were derived, two parallel directions were followed: the first to identify the unique mechanisms that were invoked in all types of stresses but were not operating under nonstress conditions; the second to identify the unique mechanisms that were invoked by each individual type of stress, but were not operating in the nonstress conditions.

### 3.5 Case 1

Two clusters were derived: C1, which included all the stress‐enriched studies (1–12 in Table 1); and C2, with all the nonstress studies (13–18 in Table 1). Given these two study clusters, two large datasets were obtained, each containing 121 genes and a number of columns equal to the sum of the samples of each study in the cluster: 413 in C1 and 344 in C2. For each study‐cluster dataset, a Gene Regulatory Network was built by applying ‘graphical lasso‐estimation of Gaussian graphical models’ (glasso) (Friedman et al., 2008) with the penalization parameter ρ = .01 (glasso is an algorithm that scales well for a large number of genes). Using the corresponding adjacency matrices of each network, it was possible to select the connections that appeared only in the stress network (unique connections).

**Figure 1:**
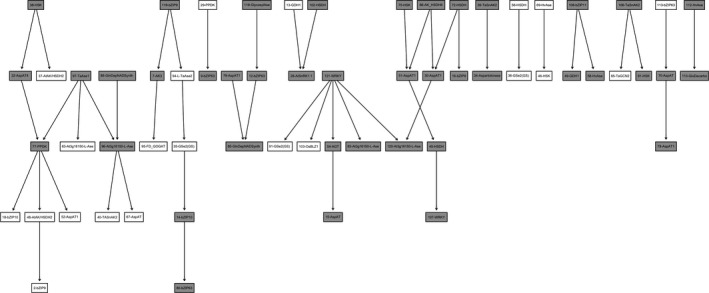
Stress‐enriched unique network. Nodes with grey background indicate genes with an internal prediction accuracy higher than 0.5 (the probability of occurring by chance is 0.333)

**Figure 2:**
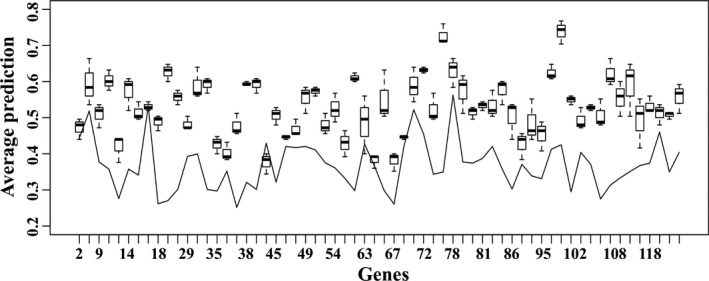
Accuracy of internal versus external prediction for genes. The boxplots in the figure indicate each gene's internal prediction, while the line indicates each gene's average external prediction

The two cluster‐derived datasets were discretized into three possible states (underregulated, normal, and overregulated), and Bayesian Networks (Heckerman, Geiger, & Chickering, 1995) were derived using hill‐climbing available in the bnlearn package (Scutari, 2010). In the process of building the structure of the networks, using the blacklist option (which allows specific links to be disallowed), the algorithm was allowed to create a connection only if this existed in the list of unique connections. At this point, the inference feature of Bayesian Networks was used to calculate the prediction accuracy (the average of correctly predicted values among the total predictions) for each gene among all studies internal to the stress‐enriched study cluster (*intra*prediction) and external to it (*inter*prediction), using the leave‐one‐out approach. Focusing on mechanisms operating in the asparagine network in wheat under stress conditions, Figure 1 shows the unique network for the stress‐enriched cluster C1. The genes highlighted in grey indicate those genes for which the internal prediction accuracy is greater or equal to 0.5. The chance of randomly predicting a gene correctly is 0.33 (given three possible states). The numbers in the nodes correspond to the Affymetrix IDs of different genes (Table S1). The mechanisms involved in asparagine metabolism include several cycles which, as a structure, require dynamic extensions in order to be modeled within Bayesian Networks. This was outside the scope of the study and these cycles therefore do not appear in the unique network in Figure 1. Nevertheless, the comparison of the internal prediction versus the external prediction (Figure 2) clearly shows how the genes involved in the stress unique network are much better predicted internally (within the stress study‐cluster C1) rather than externally (within the nonstress study‐cluster C2), and therefore can be concluded to be specifically involved in asparagine metabolism when wheat is growing under stress conditions.

### 3.6 Case 2

In order to compare each stress‐enriched study versus one cluster which contained all of the nonstress studies, 13 datasets were compiled, 12 of which corresponded to the stress‐enriched studies and 1 to a single nonstress study cluster with 344 samples, which was called *Cns*. As before, the 121 genes (Affymetrix IDs) related to asparagine metabolism were selected in each of the 12 stress‐enriched datasets. This time, however, one Gene Regulatory Network was constructed for each stress‐enriched study and one for the nonstress cluster. Because each single study comprised only a few tens of samples, glasso had to be applied with a more stringent condition in order to limit the number of false‐positive links (Friedman et al., 2008), and therefore the penalization parameter was set at ρ = .03, whereas the larger number of samples in the nonstress cluster allowed application of glasso with ρ = .001.

At this point, the process was as described in Case 1 for each combination of single study versus nonstress study cluster. Each stress‐enriched network was filtered with the nonstress network and the list of unique connections was used to build the unique networks, one for each stress‐enriched study. As expected, given the smaller number of samples in each stress‐enriched study, fewer genes presented a prediction accuracy higher than 0.5 compared with the network in Case 1, but the average intrastudy prediction was generally higher than the interstudy prediction, and where the means were similar the variance was greater externally than internally (not shown).

The relatively small number of genes with a prediction accuracy higher than 0.5 was attributable to three factors: firstly, the small number of samples involved in each stress‐enriched study; secondly, the fact that genes that are important in asparagine metabolism under stress conditions may still be related under nonstress conditions; thirdly, although the literature‐derived structure of asparagine metabolism involves several cycles, these were filtered out for the reason described above, and this would certainly have resulted in loss of information.

### 3.7 Asparagine metabolism in wheat roots under drought stress

**Figure 3:**
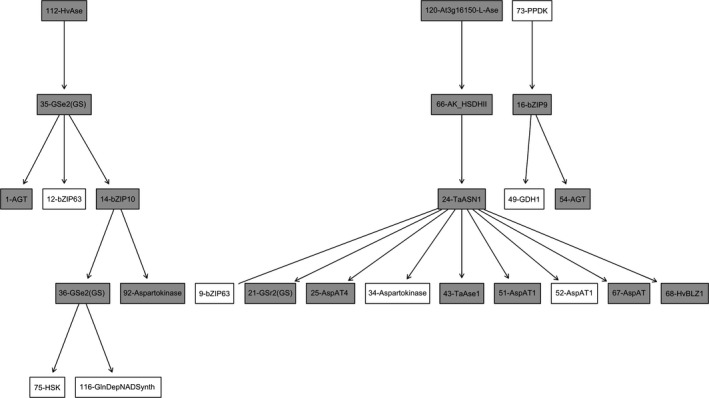
Networks based on genes involved in asparagine metabolism that are expressed only under drought stress in wheat roots. Genes are indicated by the numbers assigned in Table S1. Nodes with grey background indicate genes with an internal prediction accuracy higher than 0.5

A regulatory network of genes involved in asparagine metabolism in wheat roots specifically under drought stress was compiled using data from study E‐GEOD‐42214 (Table 1) and is presented in Figure 3. The genes shown in the figure are not expressed under normal conditions and are therefore representative only for the specific stress condition of drought. The three genes at the top of the network are genes 112 and 120 (Table 1), both encoding asparaginases, and gene 73, encoding pyruvate orthophosphate dikinase (PPDK). This is consistent with the fact that asparaginase genes have been shown to be induced in many plant species by thermal and osmotic stress, including the osmotic stress caused by drought conditions (Gaufichon et al., 2010). However, different results have been published for soybean, showing asparaginase to be induced by low temperature, ABA and NaCl but not heat shock or drought stress (Cho et al., 2007). Pyruvate orthophosphate dikinase has been shown to be induced in the roots of rice seedlings during gradual drying, cold, high salt, and water deficit response (Moons, Valcke, & Van Montagu, 1998), and its overexpression in maize has been shown to improve drought tolerance (Gu, Qiu, & Yang, 2013).

Gene 35 (glutamine synthetase; GSe1/2) follows gene 112 (asparaginase), while gene 66 (aspartate kinase; AK_HSDH I/II) follows gene 120 (asparaginase), and gene 16 (bZIP9) follows gene 73 (PPDK). Again there is consistency with what is known about these genes: glutamine synthetases have been classified previously as metabolic indicators of drought stress (Nagy et al., 2013), while aspartate kinase, although not directly associated with drought stress before, has been proposed as a sensor for activation of hyperosmotic stress (Zhu, 2002). Gene 35 (glutamine synthetase) is further connected to gene 1 (alanine‐glyoxylate aminotransferase; AGT), gene 12 (bZIP63), and gene 14 (bZIP10), then gene 14 (bZIP10) is further connected to gene 36 (glutamine synthetase; GSr 1/2) and 92 (aspartate amino transferase 2). After gene 36 (GSr 1/2) are gene 75 (homoserine kinase; HSK) and gene 116 (glutamine‐dependent NAD^(+)^ synthase). This is the first time that influences have been discovered between these genes.

In the second series of connections in Figure 3, gene 66 (aspartate kinase) is followed by gene 24 (asparagine synthetase, ASN1). This is further connected to two genes encoding transcription factors, gene 9 (bZIP63) and gene 68 (BLZ1), as well as genes 25, 51, 52, and 67 (all encoding aspartate amino transferases), gene 34 (monofunctional aspartate kinase), and gene 43 (asparaginase). As discussed above, the bZIP63 transcription factor has been shown to interact with protein kinase SnRK1 to promote expression of asparagine synthetase gene *ASN1*, which is dark induced and sugar repressed, in Arabidopsis (Baena‐González et al., 2007). The position of asparagine synthetase as a hub in the network, linking with nine other genes, is consistent with the notion of asparagine having a role not only in nitrogen transport but also as a signaling molecule, something that has been suggested by Foyer, Parry, and Noctor (2003) and Seifi, De Vleesschauwer, Aziz, and Hofte (2014).

The final part of this network comprises gene 16 (bZIP9), gene 49 (glutamate dehydrogenase; GDH), and gene 54 (alanine‐glyoxylate aminotransferase; AGT). The link among bZIP9, GDH, and AGT has not been described previously, but both AGT and glutamate‐glyoxylate aminotransferase have been shown to be inhibited by hypoxia (Ricoult, Echeverria, Cliquet, & Limami, 2006).

### 3.8 Asparagine metabolism in wheat leaves under drought stress

**Figure 4:**
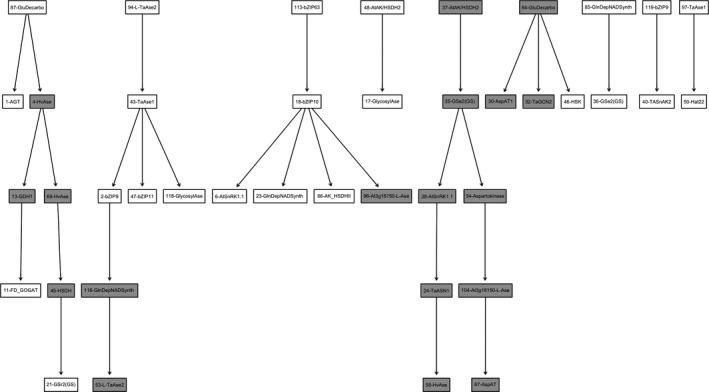
Networks based on genes involved in asparagine metabolism that are expressed only under drought stress in wheat leaves. Genes are indicated by the numbers assigned in Table S1. Nodes with grey background indicate genes with an internal prediction accuracy higher than 0.5

Figure 4 represents a network of genes involved in asparagine metabolism that are expressed in wheat leaves under drought stress, and was compiled using data from study E‐GEOD‐31759 (Table 1). This figure could be separated into nine parts. The first one starts with gene 87 (glutamate decarboxylase) followed by gene 1 (alanine‐glyoxylate aminotransferase; AGT) and gene 4 (asparaginase). It is notable that asparaginase and AGT genes also featured in Figure 3, while the role of glutamate decarboxylase is consistent with its involvement in drought stress responses (it synthesizes γ‐amino butyric acid (GABA) from glutamate (Mohammadi, Kav, & Deyholos, 2007) and GABA may function as an osmoprotectant under stress conditions). The genes that follow gene 4 (asparaginase) are as follows: gene 13 (glutamate dehydrogenase; GDH1) and gene 69 (asparaginase). Gene 13 (GDH1) is connected to gene 11 (Fd_GOGAT). On the other side of this series of connections, the genes following genes 4 and 69 (asparaginase) are gene 45 (homoserine dehydrogenase; HSDH) and gene 21 (glutamine synthetase; GSr). Note that gene 69 (asparaginase) arises twice to avoid a closed link.

Again, there are consistencies with the results of previous experimental studies. Overexpression of an *E. coli GDH* gene, for example, has been shown to improve drought tolerance in maize (Lightfoot et al., 2007), while Fd‐GOGAT gene expression has been shown to increase in *Lotus corniculatus* under drought stress (Borsani, Diaz, & Monza, 1999). The link between stress, GS, and GDH has also been investigated in wheat (Sairam, 1994): drought‐tolerant genotypes were shown to have higher activities of both these enzymes and of nitrate reductase under drought stress, compared with drought‐sensitive genotypes. This was associated with the maintenance of higher relative water content, membrane stability, chlorophyll content, and photosynthesis.

The second part of the network starts with gene 94 (asparaginase) followed by gene 43 (also asparaginase). A link between these two asparaginase genes has been suggested in a study in which severe drought significantly decreased the activities of key enzymes of nitrogen anabolism, such as nitrate reductase, glutamine synthetase, and glutamate dehydrogenase, but increased the activities of enzymes involved in nitrogen catabolism, including asparaginase and endopeptidase (Xu & Zhou, 2006). Following the two asparaginase genes are gene 2 (bZIP9), gene 47 (bZIP11), and gene 118 (glycosyl asparaginase; an enzyme that hydrolyzes the β‐N‐glycosidic bond between asparagine and N‐acetylglucosamine in asparagine‐linked glycans). After gene 2 (bZIP9) are gene 116 (glutamine‐dependent NAD^(+)^ synthetase) followed by gene 53 (another asparaginase). As well as regulating asparagine synthetase‐1 and proline dehydrogenase‐2 gene expression (Hanson, Hanssen, Wiese, Hendriks, & Smeekens, 2008), bZIP11 is a regulator of trehalose metabolism (Hanson et al., 2008; Ma et al., 2011), and trehalose metabolism is now known to play a key role in drought stress tolerance (Lawlor & Paul, 2014).

The third part of the network in Figure 4 comprises gene 113 (bZIP63) followed by gene 18 (bZIP10), with bZIP10 then affecting gene 6 (SnRK1.2), gene 23 (glutamine‐dependent NAD^(+)^ synthetase), gene 66 (aspartate kinase homoserine dehydrogenase), and gene 96 (asparaginase). bZIP63 has been shown to be involved in SnRK1‐induced responses to energy limitation, and to be an important node of the glucose‐ABA interaction network (Matiolli et al., 2011), while bZIP10 shuttles between the nucleus and the cytoplasm and binds consensus G‐ and C‐box DNA sequences. It has been reported to be retained outside the nucleus by LSD1, a protein protecting Arabidopsis from death caused by oxidative stress signals (Kaminaka et al., 2006). bZIP10 has not previously been linked directly to drought, but it has been suggested to have roles in stress responses and, more specifically, in amino acid metabolism, and sink‐specific gene expression (Kaminaka et al., 2006).

The fourth part of the network comprises gene 48 (homoserine dehydrogenase) and gene 17 (glycosyl asparaginase). The fifth part starts with gene 37 (aspartate kinase) followed by gene 35 (glutamine synthetase; GSe). It then splits into two: gene 28 (SnRK1) and gene 34 (aspartokinase). SnRK1 affects gene 24 (ASN1), followed by gene 58 (asparaginase), while in the other branch, gene 34 (aspartokinase) is followed by gene 104 (asparaginase) and finally gene 67 (AspAT). This part of the network represents the direct effect of drought stress on asparagine metabolism described by Lea et al. (2007). As discussed above, the expression of the Arabidopsis *AtASN1* gene has been shown to increase when SnRK1 is overexpressed in transgenic plants (Baena‐González et al., 2007). Furthermore, the promoter of *AtASN1* contains a G‐Box sequence known to be bound by a bZIP transcription factor (Delatte et al., 2011). The link between asparaginase and asparagine synthetase may occur because they are connected *via* accumulation/remobilization of asparagine: when asparagine is accumulated as a result of drought stress, it may be catabolized by asparaginase to supply nitrogen for the synthesis of other amino acids (Grant & Bevan, 1994; Sotero‐Martins, da Silva Bon, & Carvajal, 2003).

The sixth part of the network starts with gene 64 (glutamate decarboxylase), followed by gene 30 (AspAT), gene 32 (GCN2), and gene 46 (homoserine kinase). These three genes are coexpressed during amino acid biosynthesis. A link between aspartate amino transferase and GCN2 was shown by Zhang et al. (2008) using an Arabidopsis mutant lacking a functional GCN2.

Each of the next three groups consists of only two genes: gene 85 (glutamine‐dependent NAD^(+)^ synthetase) connected to gene 36 (glutamine synthetase; GSe1/2); gene 119 (bZIP9) connected to gene 40 (SnRK1); and gene 97 (asparaginase) connected to gene 50 (HAT22). The only one of these links to be reported previously was that between HAT22 and asparaginase (Thum et al., 2008).

### 3.9 Influence of nutrient supply on asparagine metabolism: elucidation of networks operating under conditions of sulfur and nitrogen deficiency

**Figure 5:**
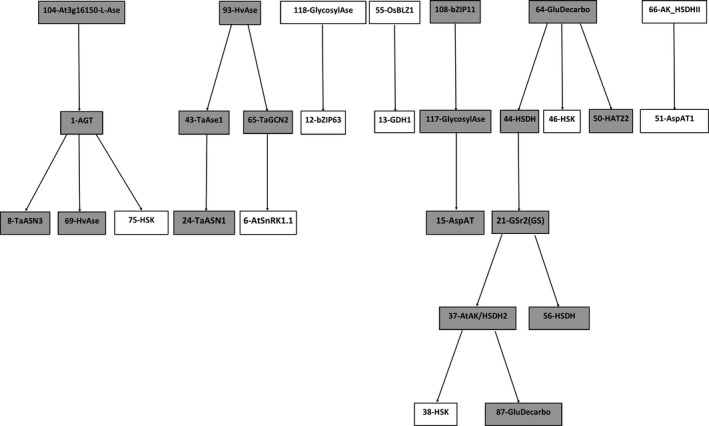
Networks based on genes involved in asparagine metabolism that are expressed only under conditions of sulfur and nitrogen deficiency in wheat leaves. Genes are indicated by the numbers assigned in Table S1. Nodes with grey background indicate genes with an internal prediction accuracy higher than 0.5

Free asparagine and total free amino acids have been shown to accumulate to greatly increased levels in wheat in response to sulfur deficiency (Curtis et al., 2009; Granvogl et al., 2007; Muttucumaru et al., 2006). It has been suggested that free asparagine is used as a nitrogen store under these conditions, making the balance of sulfur and nitrogen availability an important factor in preventing asparagine accumulation (Curtis, Postles, & Halford, 2014). A network was therefore constructed to represent responses of genes involved in asparagine metabolism in leaves of winter wheat under conditions of sulfur and nitrogen deficiency (Figure 5), using data from a transcription profiling time series (Table 1, study: E‐MEXP‐1415). The network consists of 28 genes, with the first part starting with gene 104 (asparaginase), followed by gene 1 (alanine‐glyoxylate aminotransferase; AGT), then three further genes: gene 8 (asparagine synthetase‐3; TaASN3), gene 69 (asparaginase), and gene 75 (homoserine kinase; HSK). The second part of the network starts with gene 93 (asparaginase; HvAse), followed by gene 43 (asparaginase) and 65 (TaGCN2). Further to these, respectively, are genes 24 (TaASN1) and 6 (SnRK1). The study therefore suggests that *TaASN1* and *TaASN3* are both affected by nutrient availability, consistent with the findings of Gao et al. (2016).

The next two sections are very short: gene 118 (glycosyl asparaginase) affects gene 12 (bZIP63), and gene 55 (transcription factor BLZ1) affects gene 13 (glutamate dehydrogenase 1/2; GDH1/2). The *BLZ1* gene is expressed during early endosperm development in barley, as well as in roots and leaves (Vicente‐Carbajosa, Onate, Lara, Diaz, & Carbonero, 1998), and has been associated with flowering time and plant height (Haseneyer et al., 2010). BLZ1 binds to the N‐motif, a promoter element with the nucleotide sequence ATGAGTCATC that was first characterized as a nitrogen‐responsive element in cereal seed storage protein genes (reviewed by Halford & Shewry, 2007) but is also found in the promoter of *TaASN1* and *ASN1* genes from multiple cereal species (Gao et al., 2016).

The next series starts with gene 108 (bZIP11), followed by gene 17 (glycosyl asparaginase) and gene 15 (aspartate amino transferase; AspAT). The next section is larger, comprising nine genes in total and starting with gene 64 (glutamate decarboxylase), then three genes: gene 44 (homoserine dehydrogenase), gene 46 (homoserine kinase; HSK), and gene 50 (HAT22). After gene 44 (homoserine dehydrogenase) comes gene 21 (glutamine synthetase; GSr1/2), then two more genes: gene 37 (aspartate kinase‐homoserine dehydrogenase‐2) and gene 56 (homoserine dehydrogenase; HSDH). The series finishes with gene 38 (homoserine kinase; HSK) and gene 87 (glutamate decarboxylase), and therefore contains the glutamine synthetase, glutamate and glutamate decarboxylase loop. All of these genes have been shown to be affected by nitrogen availability (Forde & Lea, 2007).

The final part of the network consists of two genes: gene 66 (aspartate kinase; AK‐HSDH) and gene 51 (aspartate amino transferase; AspAT1). The expression of both these genes has been shown to increase in Arabidopsis upon sulfur starvation (Nikiforova et al., 2006).

## 4 DISCUSSION

This study represents the construction of a detailed and comprehensive asparagine metabolism network. The network comprises stimuli, enzymes, genes, and small molecules, including asparagine itself, glutamine, aspartate, and glutamate, and energy molecules, including ATP, ADP, and NADH. The network was hand curated using data from existing databases and literature from a range of species, but was applied to the analysis of transcriptomic data from wheat plants to identify genes involved in asparagine metabolism under conditions of drought stress and nutrient deficiency. This also served as a validation exercise because it identified interactions between genes in the network.

Previous modeling studies of nitrogen assimilation, amino acid, and sugar metabolism have been much narrower in scope. The first nitrogen assimilation network was published by Champigny (1995), but asparagine and aspartate were excluded. Research on nitrogen assimilation at that time focused mainly on glutamine and glutamate (Sechley, Yamaya, & Oaks, 1992). In carbon metabolism, Wienkoop et al. (2008) published an investigation of the combined covariance structure of metabolite and protein dynamics in the systemic response to abiotic stress in wild‐type Arabidopsis and a mutant with a starch deficiency phenotype caused by a dysfunctional phosphodismutase gene. Independent component analysis was used to reveal phenotype classifications resolving genotype‐dependent response effects to temperature treatment, and genotype‐independent general temperature compensation mechanisms (Wienkoop et al., 2008). Modeling approaches, including Boolean logic, have also been used for a systematic exploration of the interactions between light and sugar signaling in the regulation of asparagine synthetase and glutamine synthetase in Arabidopsis (Thum et al., 2008). However, the network constructed in this study far exceeds anything on asparagine metabolism in the literature.

It is clear from the networks that were developed in this study that the expression of many genes involved in asparagine metabolism is altered in response to stress conditions and nutrient availability. Some of the responses and relationships identified in the construction of the networks are well documented in the literature, but others have not been described in any detail and the nature of the relationship remains unknown. Potentially important interactions for wheat grain development and composition that require further study include that between GCN2, SnRK1, and asparagine synthetase, while detailed knowledge is also lacking on the potentially important role of HAT22 in regulating asparagine metabolism, the influence of asparaginase, glutamate dehydrogenase, and homoserine dehydrogenase on asparagine metabolism under drought stress, and the role of bZIP transcription factors in general responses to drought stress. Nevertheless, the network could be used to predict the response of genes in asparagine metabolism to stress or other stimuli, and identifies sets of genes whose expression defines particular stresses. It could also provide a basis for developing strategic genetic interventions to manipulate asparagine concentration, and for the application of other modeling techniques to this crucial area of plant metabolism.

## CONFLICT OF INTEREST

None declared.
