# Supplementary figures and images for: Construction of a network describing asparagine metabolism in plants and its application to the identification of genes affecting asparagine metabolism in wheat under drought and nutritional stress
Source: Food Energy Secur. 2018 Feb 25;7(1):e00126. doi: 10.1002/fes3.126 (PMC5993343; doi:10.1002/fes3.126)

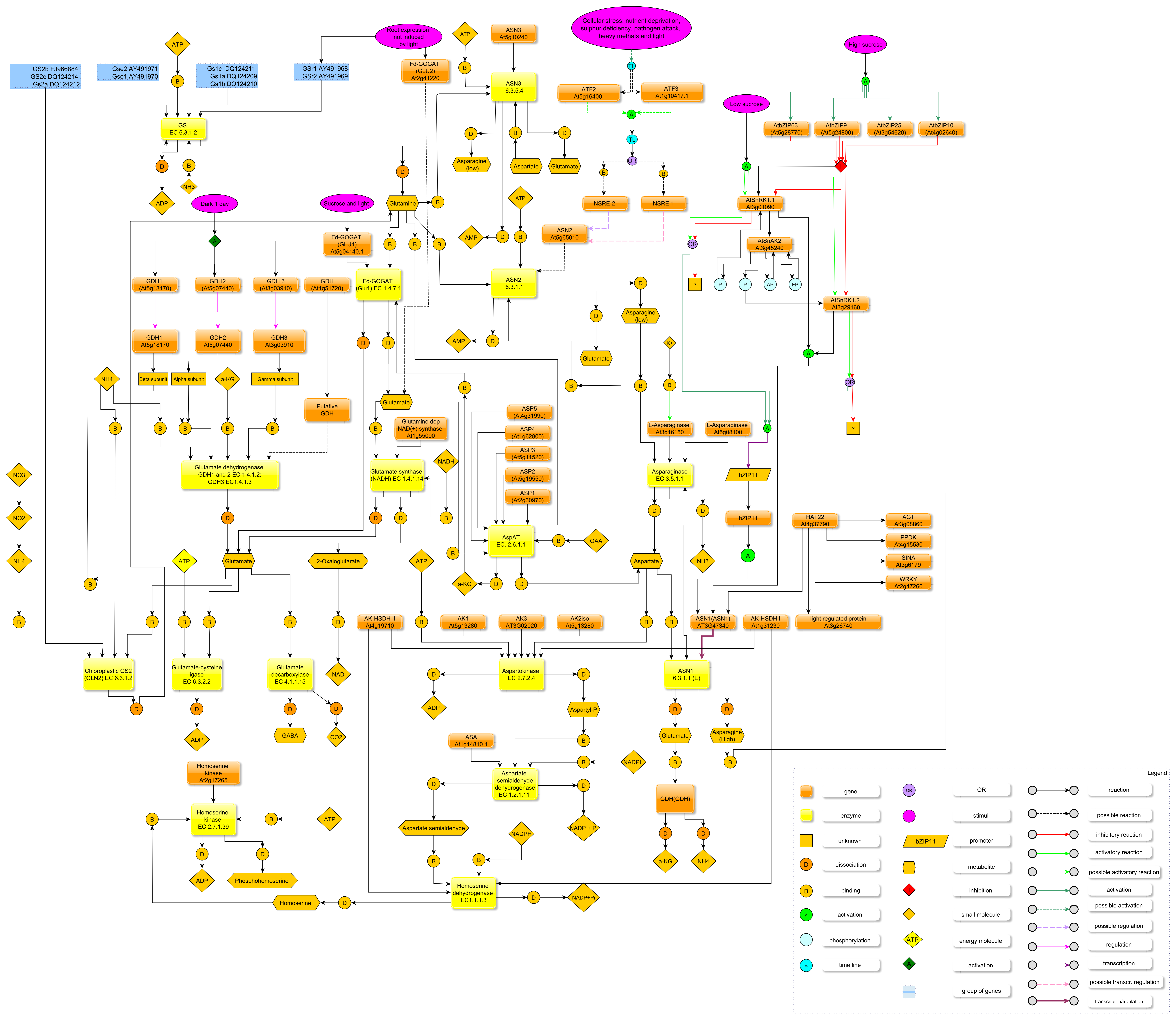

Supplement: Supplementary file 1 [file FES3-7-na-s001.pdf]
