# Supplementary material for: Construction of a network describing asparagine metabolism in plants and its application to the identification of genes affecting asparagine metabolism in wheat under drought and nutritional stress
Source: Food Energy Secur. 2018 Feb 25;7(1):e00126. doi: 10.1002/fes3.126 (PMC5993343; doi:10.1002/fes3.126)
Supplement: Supplementary file 2 [file FES3-7-na-s002.pdf]

Construction of mathematical networks describing asparagine metabolism that predict changes in gene expression in response to stress

Tanya Y. Curtis[1*], Valeria Bo[2**], Allan Tucker[2] and Nigel G. Halford[1]

[1]Plant Science Department, Rothamsted Research, Harpenden, Hertfordshire AL5 2JQ, United Kingdom

[2]College of Engineering, Design and Physical Sciences, Brunel University London, Uxbridge, Middlesex UB8 3PH, UK

[*]Author for correspondence. Current address: Dr Tanya Curtis, Curtis Analytics Ltd, Daniel Hall Building, Rothamsted RoCRE, Harpenden AL5 2JQ, United Kingdom. Tel: 07766748793; Email: curtistanya4@gmail.com

[**]Current address: Cancer Research UK Cambridge Institute, University of Cambridge, Li Ka Shing Centre, Robinson Way, Cambridge CB2 0RE, UK.

Supplementary Table S1

List and annotation of genes covered by the network.

Gene number	Gene name	Target ID	
		Name assigned to transcript	Sequence annotation
1	AGT	Ta.10890.1.S1_at	
2	bZIP9	Ta.12251.1.S1_x_at	262537_s_at
3	TaGCN2	Ta.12655.1.S1_at	
4	HvAse	Ta.12746.1.S1_at	257588_x_at
5	L-TaAse2	Ta.12881.1.A1_at	251101_at
6	AtSnRK1.1	Ta.1312.1.S1_at	
6	SnRK1.2	Ta.1312.1.S1_at	
6	TaSNF1_ 1.3	Ta.1312.1.S1_at	
7	AK3	Ta.13407.1.S1_at	
7	AtAK2	Ta.13407.1.S1_at	
7	AtAK1	Ta.13407.1.S1_at	
7	AtAK/HSDH2	Ta.13407.1.S1_at	
8	TaASN3	Ta.13987.1.S1_at	250484_at
9	bZIP63	Ta.14216.1.S1_at	
10	L-TaAse2	Ta.14364.2.S1_at	251101_at
11	FD_GOGAT	Ta.15778.1.S1_at	245701_at
11	FD_GOGAT	Ta.15778.1.S1_at	266365_at
12	bZIP63	Ta.15865.1.A1_at	
13	aGDH subunit	Ta.1870.1.S1_a_at	259346_at
13	GDH2	Ta.1870.1.S1_a_at	250580_at
13	bGDH subunit	Ta.1870.1.S1_a_at	
13	GDH2	Ta.1870.1.S1_a_at	250580_at
14	bZIP10	Ta.19597.1.S1_at	251134_at
15	AspAT	Ta.20913.1.S1_a_at	263429_at
16	bZIP9	Ta.21094.1.S1_at	
17	GlycosylAse	Ta.21112.1.S1_x_at	
18	bZIP10	Ta.23671.1.S1_x_at	263907_at
19	AtSnRK1.1	Ta.24092.1.A1_at	
19	TaSNF	Ta.24092.1.A1_at	
19	SnRK1.2	Ta.24092.1.A1_at	
19	TaSNF1_ 1.3	Ta.24092.1.A1_at	249450_at
20	HSK	Ta.2419.3.S1_at	
21	GSr2(GS)	Ta.24508.1.S1_x_at	258160_at
21	GSr1(GS)	Ta.24508.1.S1_x_at	258160_at
22	AspAT4	Ta.24982.1.S1_at	262646_at
22	AspAT3	Ta.24982.1.S1_at	245951_at
22	AspAT	Ta.24982.1.S1_at	262646_at
23	GlnDepNADSynth	Ta.26779.1.A1_at	
24	TaASN1	Ta.2690.1.S1_at	250484_at
24	TaASN3	Ta.2690.1.S1_at	247218_at

25	AspAT4	Ta.26933.1.S1_at	
25	AspAT	Ta.26933.1.S1_at	253481_at
26	AspAT	Ta.2703.1.S1_at	259199_at
27	HvAse	Ta.27208.1.S1_at	
28	AtSnRK1.1	Ta.27319.1.S1_at	259319_at
28	AtSnRK1.1	Ta.27319.1.S1_at	258221_at
28	TaSNF	Ta.27319.1.S1_at	259319_at
28	SnRK1.2	Ta.27319.1.S1_at	258221_at
28	SnRK1.2	Ta.27319.1.S1_at	259319_at
28	TaSNF1_ 1.3	Ta.27319.1.S1_at	258221_at
29	PPDK	Ta.27771.1.S1_at	245528_at
30	AspAT1	Ta.28228.1.S1_at	262645_at
31	AspAT1	Ta.28228.2.S1_at	
32	TaGCN2	Ta.28336.1.S1_x_at	
33	AGT	Ta.28441.1.S1_at	
34	Aspartokinase	Ta.2845.1.S1_at	258977_s_at
34	AK3	Ta.2845.1.S1_at	250291_at
34	AtAK2	Ta.2845.1.S1_at	258977_s_at
34	AK2	Ta.2845.1.S1_at	250291_at
34	AtAK2	Ta.2845.1.S1_at	258977_s_at
34	AtAK2	Ta.2845.1.S1_at	250291_at
34	AK1	Ta.2845.1.S1_at	250291_at
34	AtAK1	Ta.2845.1.S1_at	250291_at
34	AtAK1	Ta.2845.1.S1_at	258977_s_at
34	AtAK/HSDH2	Ta.2845.1.S1_at	258977_s_at
34	AtAK/HSDH2	Ta.2845.1.S1_at	250291_at
35	GSe2(GS)	Ta.2870.1.S1_at	
35	GSe1(GS)	Ta.2870.1.S1_at	
36	GSe2(GS)	Ta.2870.2.S1_at	256524_at
36	GSe1(GS)	Ta.2870.2.S1_at	256524_at
37	AtAK/HSDH2	Ta.28955.1.S1_at	
37	AK_HSDHII	Ta.28955.1.S1_at	
37	AK_HSDH1	Ta.28955.1.S1_at	
37	BAK/HSDH2	Ta.28955.1.S1_at	
38	HSK	Ta.3019.1.S1_at	248106_at
39	TaSnAK2	Ta.3031.2.S1_at	247635_at
39	AtSNAK2	Ta.3031.2.S1_at	247635_at
39	AtSNAK2	Ta.3031.2.S1_at	247635_at
39	AtSnAK1	Ta.3031.2.S1_at	247635_at
40	TASnAK2	Ta.30734.2.A1_at	247635_at
40	AtSnAK1	Ta.30734.2.A1_at	
40	AtSNAK2	Ta.30734.2.A1_at	

41	AGT	Ta.3233.1.A1_at	264836_at
42	HvAse	Ta.3373.1.S1_at	
43	TaAse1	Ta.3504.2.A1_at	258338_at
43	L-TaAse2	Ta.3504.2.A1_at	258338_at
43	At3g16150-L-Ase	Ta.3504.2.A1_at	258338_at
43	HvAse	Ta.3504.2.A1_at	258338_at
44	HSDH	Ta.4073.2.S1_at	260518_at
46	HSK	Ta.454.1.S1_at	252873_at
47	bZIP11	Ta.4604.1.S1_at	
48	AtAK/HSDH2	Ta.4812.1.S1_at	254535_at
48	AK_HSDHII	Ta.4812.1.S1_at	263696_at
48	AK_HSDHI	Ta.4812.1.S1_at	263696_at
48	AK_HSDH1	Ta.4812.1.S1_at	254535_at
48	BAK/HSDH2	Ta.4812.1.S1_at	254535_at
48	BAK/HSDH2	Ta.4812.1.S1_at	263696_at
48	BAK/HSDH1	Ta.4812.1.S1_at	263696_at
48	BAK/HSDH1	Ta.4812.1.S1_at	254535_at
49	GDH1	Ta.5091.3.S1_x_at	250032_at
49	aGDH subunit	Ta.5091.3.S1_x_at	250032_at
49	aGDH subunit	Ta.5091.3.S1_x_at	259346_at
49	bGDH subunit	Ta.5091.3.S1_x_at	250032_at
49	GDH2	Ta.5091.3.S1_x_at	250032_at
50	Hat22	Ta.5195.1.S1_at	266824_at
51	AspAT1	Ta.5314.1.S1_x_at	267151_at
52	AspAT1	Ta.5314.3.S1_a_at	
53	L-TaAse2	Ta.5446.1.S1_x_at	
53	At3g16150-L-Ase	Ta.5446.1.S1_x_at	
54	AGT	Ta.568.1.S1_at	263350_at
55	OsBLZ1	Ta.5909.1.S1_at	
56	HSDH	Ta.6194.1.S1_at	265503_at
57	TaASN2	Ta.6223.1.S1_at	**252415_at**
57	TaASN2	Ta.6223.1.S1_at	**250484_at**
58	HvAse	Ta.6466.1.S1_at	263872_at
59	bZIP11	Ta.6518.1.S1_at	252969_at
60	GlycosylAse	Ta.6828.1.S1_a_at	267627_at
61	GlycosylAse	Ta.6828.1.S1_at	
62	HSK	Ta.7110.1.S1_at	
63	At3g16150-L-Ase	Ta.7165.1.A1_at	
64	GluDecarbo	Ta.7341.1.S1_at	261970_at
65	TaGCN2	Ta.7966.1.S1_a_at	246294_at
66	AK_HSDHII	Ta.8999.1.A1_at	
66	AK_HSDHI	Ta.8999.1.A1_at	

66	BAK/HSDH2	Ta.8999.1.A1_at	
66	BAK/HSDH1	Ta.8999.1.A1_at	
67	AspAT	Ta.9466.2.S1_a_at	
68	HvBLZ1	Ta.9612.1.S1_at	266738_at
69	HvAse	Ta.9752.1.S1_at	265093_at
70	AspAT	TaAffx.106456.1.S1_at	
71	TaGCN2	TaAffx.108357.1.S1_at	251520_at
72	HSDH	TaAffx.108979.1.S1_at	
73	PPDK	TaAffx.111397.1.S1_at	267290_at
74	GDH2	TaAffx.112540.1.S1_at	
75	HSK	TaAffx.112795.1.S1_at	
76	HSK	TaAffx.113257.1.S1_at	255199_at
77	PPDK	TaAffx.113765.1.S1_at	
77	AGT	TaAffx.113765.1.S1_at	
78	AspAT1	TaAffx.113950.2.S1_at	
79	AspAT1	TaAffx.113950.2.S1_x_at	263089_at
80	bZIP63	TaAffx.114167.1.S1_at	247360_at
81	HSK	TaAffx.117163.1.S1_at	247909_at
82	HvAse	TaAffx.118887.1.S1_at	259176_at
82	GlycosylAse	TaAffx.118887.1.S1_at	259176_at
83	At3g16150-L-Ase	TaAffx.119291.1.S1_at	266884_at
84	Hat22	TaAffx.119940.2.S1_at	
85	GlnDepNADSynth	TaAffx.128473.2.A1_at	
86	GlnDepNADSynth	TaAffx.128473.2.A1_x_at	245014_at
87	GluDecarbo	TaAffx.129066.1.S1_at	250090_at
88	At3g16150-L-Ase	TaAffx.130104.3.S1_at	255684_at
89	GSr2(GS)	TaAffx.143995.3.A1_at	
89	GSr1(GS)	TaAffx.143995.3.A1_at	
90	GSe2(GS)	TaAffx.143995.4.A1_at	
90	GSe1(GS)	TaAffx.143995.4.A1_at	
91	GSe2(GS)	TaAffx.143995.4.S1_s_at	249581_at
91	GSe1(GS)	TaAffx.143995.4.S1_s_at	249581_at
92	Aspartokinase	TaAffx.16105.1.A1_at	
92	AtAK2	TaAffx.16105.1.A1_at	
92	AtAK2	TaAffx.16105.1.A1_at	
92	AtAK1	TaAffx.16105.1.A1_at	
92	AtAK/HSDH2	TaAffx.16105.1.A1_at	
93	HvAse	TaAffx.20511.1.S1_at	
93	GlycosylAse	TaAffx.20511.1.S1_at	
94	L-TaAse2	TaAffx.22667.1.S1_at	261499_at
94	At3g16150-L-Ase	TaAffx.22667.1.S1_at	261499_at
95	FD_GOGAT	TaAffx.22799.1.S1_at	266365_at

96	At3g16150-L-Ase	TaAffx.24735.1.S1_at	247530_at
97	TaAse1	TaAffx.25115.3.S1_at	250547_at
97	At3g16150-L-Ase	TaAffx.25115.3.S1_at	250547_at
97	HvAse	TaAffx.25115.3.S1_at	250547_at
98	HSDH	TaAffx.25294.1.S1_s_at	
99	bZIP11	TaAffx.25548.1.S1_at	263414_at
100	HSK	TaAffx.26750.1.S1_at	264855_at
101	AspAT	TaAffx.30234.1.S1_at	
102	HSDH	TaAffx.30631.1.S1_at	246027_at
103	OsBLZ1	TaAffx.31672.1.S1_at	260625_at
104	At3g16150-L-Ase	TaAffx.32271.1.S1_s_at	262902_x_at
105	WRKY	TaAffx.44653.1.A1_at	
106	TaSnAK2	TaAffx.51251.1.S1_at	252616_at
106	AtSNAK2	TaAffx.51251.1.S1_at	252616_at
106	AtSnAK1	TaAffx.51251.1.S1_at	252616_at
106	AtSNAK2	TaAffx.51251.1.S1_at	252616_at
107	WRKY	TaAffx.52006.1.S1_at	
108	bZIP11	TaAffx.56532.1.S1_at	
109	AspAT3	TaAffx.57918.1.S1_at	250385_at
110	GluDecarbo	TaAffx.59890.1.S1_at	
111	OsBLZ1	TaAffx.62718.1.S1_at	252149_at
112	HvAse	TaAffx.67800.1.A1_at	257273_at
113	bZIP63	TaAffx.70442.1.A1_s_at	246399_at
114	At3g16150-L-Ase	TaAffx.79563.1.S1_at	255448_at
115	WRKY	TaAffx.82096.1.S1_at	249387_at
116	GlnDepNADSynth	TaAffx.83639.1.S1_at	251719_at
117	HvAse	TaAffx.84887.1.S1_at	
118	GlycosylAse	TaAffx.86537.1.S1_at	252246_at
119	bZIP9	TaAffx.90174.1.S1_at	248207_at
120	At3g16150-L-Ase	TaAffx.9114.1.S1_at	
121	WRKY	TaAffx.91377.1.S1_at	249770_at
