# Supplementary material for: Construction of a network describing asparagine metabolism in plants and its application to the identification of genes affecting asparagine metabolism in wheat under drought and nutritional stress
Source: Food Energy Secur. 2018 Feb 25;7(1):e00126. doi: 10.1002/fes3.126 (PMC5993343; doi:10.1002/fes3.126)
Supplement: Supplementary file 3 [file FES3-7-na-s003.docx]

**Figure S1.** Network describing asparagine metabolism, created with yED Graph Editor Version 3.2.0.1 (yWorks, Tübingen Germany) (free download from https://www.yworks.com/downloads#yEd)
